# Prediction of Short-Term Stock Price Trend Based on Multiview RBF Neural Network

**DOI:** 10.1155/2021/8495288

**Published:** 2021-11-28

**Authors:** Bailin Lv, Yizhang Jiang

**Affiliations:** ^1^School of Artificial Intelligence and Computer Science, Jiangnan University, 1800 Lihu Avenue, Wuxi 214122, Jiangsu, China; ^2^Jiangsu Key Laboratory of Media Design and Software Technology, 1800 Lihu Avenue, Wuxi 214122, Jiangsu, China

## Abstract

Stock price prediction is important in both financial and commercial domains, and using neural networks to forecast stock prices has been a topic of ongoing research and development. Traditional prediction models are often based on a single type of data and do not account for the interplay of many variables. This study covers a radial basis neural network modeling technique with multiview collaborative learning capabilities for incorporating the impacts of numerous elements into the prediction model. This research offers a multiview RBF neural network prediction model based on the classic RBF network by integrating a collaborative learning item with multiview learning capabilities (MV-RBF). MV-RBF can make full use of both the internal information provided by the correlation between each view and the distinct characteristics of each view to form independent sample information. By using two separate stock qualities as input feature information for trials, this study proves the viability of the multiview RBF neural network prediction model on a real data set.

## 1. Introduction

Predicting the fluctuation trend of stock prices plays an extremely important role in asset pricing, investment decision-making, risk management, and market supervision. Through the study of the autocorrelation function, power spectral density, and fluctuation range of stock prices, it is found that there are hidden long-term price linear trends and low-frequency periodic fluctuations in stock prices. This theoretically proves that stock prices that exhibit randomness and unpredictability at the micro level have overall certainty and predictability at the macro level [1]. Therefore, the prediction of stock price trends through machine learning and other methods has become an important topic nowadays.

The BP neural network is the most extensively used model for stock price prediction. White et al. were the first to use the BP neural network to forecast stock prices [2], and numerous improved approaches based on the BP neural network followed [3]. The BP neural network, on the other hand, is readily stuck in local minimums, causing the model's prediction impact to deteriorate. By improving the SVM algorithm, Huang and Chen overcome to a certain extent the problem that the SVM algorithm can only obtain specific prediction values but cannot predict the trend of stock changes [4]. In addition, the choice of the kernel function of the SVM algorithm will also have a direct impact on the prediction accuracy. The LSTM algorithm can handle time series data problems well, but it may fall into a local optimal solution, and it has problems such as lag in prediction [5]. In addition to resolving the LSTM model's weaknesses, LSTM is frequently merged with other models to produce a hybrid model in order to increase LSTM's prediction performance [6].

All of the aforementioned model experiments are based on a single data viewpoint (view) [7]. Changes in stock prices, on the other hand, are influenced by a range of circumstances, which will eventually be represented in the stock's different characteristic data, such as the starting price, highest price, closing price, ups and downs, and trading volume. When compared to using single attribute stock data as experimental data, combining data from multiple attributes to uncover the relationship between distinct attribute data without compromising the independence of each attribute data would certainly improve the model's generalization capabilities. This work presents a multiview collaborative learning technique [8] to attain this aim. A function with collaborative learning capacity is introduced based on the construction of a matching model for each view, allowing it to fully utilize the related information between the view sample data sets [9]. Multiview learning seeks to train one function to represent each view and then optimize all of the functions at the same time to increase generalization performance [10].

The RBF NN is used as the activation function in the RBF neural network's hidden layer to execute a fixed nonlinear transformation. The output layer of the RBF neural network is linearly integrated in the new space, mapping the low-dimensional input space to the new high-dimensional output space [11–13]. The linear property of such an output unit makes RBF neural network parameter change straightforward, and there is no local minimum difficulty. RBF neural network is also a useful basis for constructing multiview collaborative learning tasks, because it has strong multidimensional nonlinear mapping skills, generalization capabilities, and parallel information processing capabilities [14, 15].

The development of the field of artificial intelligence stems from the desire to give computers a way of thinking similar to human cognition. The core idea of the multiview learning method also hopes to include more effective information and explore the relevance and independence between views [16, 17]. The data of various characteristics represent the effect of numerous elements on the stock in the process of forecasting the trend of stock price variations, and the influence of different attributes on the final outcome is certain to be different. As a result, we must examine the degree of effect of diverse viewpoints on the final output in the multiview learning process [18]. Such a decision-making process is similar to our human thinking and decision-making process, which is also the advantage of multiview collaborative learning. In the future scenario of intelligent life and the interconnection of all things, the use of a single data source for experimental analysis cannot meet actual needs. At that point, we will evaluate the substance of the present scene based on the data information produced by different data sources. The application requirements are similar to those discussed in this paper's multiview learning approach. As a result, we have cause to assume that multiview learning technology, which incorporates many data sources, will be an area of neural network research that cannot be overlooked in the future.

In this paper's experimental effort, we start with the question, “How can we enhance the accuracy of stock price prediction using existing approaches even more?” By reading the relevant literature in recent years, we have focused on the multiview direction, because the multiview thinking is a more feasible method in both cognition and theory. Next, our focus is to find multiple perspectives on stock data. We discovered that stock data is made up of several different types of information, including the highest price, lowest price, opening price, and closing price. More complicated data gathering demonstrates that multiview approaches may be used to analyze stock data. Another problem we encountered is what kind of algorithm can be used to capture the connection between different perspective data at the same time without destroying the independence of a single perspective data. After continuous attempts and improvements, we construct an objective function with collaborative learning ability. Simultaneously, the Lagrangian multiplier is applied to simplify the functional formula, which is then turned into a quadratic programming issue according to Lagrangian optimization theory. Finally, we tested the method's viability using numerous data sets in experiments.

The rest of this paper is laid out as follows. The radial basis neural network and the stock data processing approach are introduced in Section 2.  Section 3 offers our multiview RBF classification model and builds a multiview learning framework. The experimental results are presented in Section 4. The conclusion is reached in Section 5.

## 2. Related Work

In this part, we will go through stock data processing procedures including shifting windows, data normalization, and other stages. The radial basis function neural network (RBF NN) is then introduced, along with related improvement strategies, to build the theoretical and technological groundwork for the creation of multiview models.

### 2.1. Stock Data Processing

The original stock data used in this paper comes from Lvjing real estate (ID 000502), which includes information on ten different attributes such as transaction date, opening price, closing price, highest price, lowest price, yesterday's closing price, change amount, change range, trading volume, and turnover. Except for the transaction date in the original data, other stock data can be regarded as dynamic time series. For such data, a time series analysis method is usually used. The method emphasizes that a region is continuously observed and calculated for a short period time to extract relevant features and analyze its change process.

**Figure 1:**
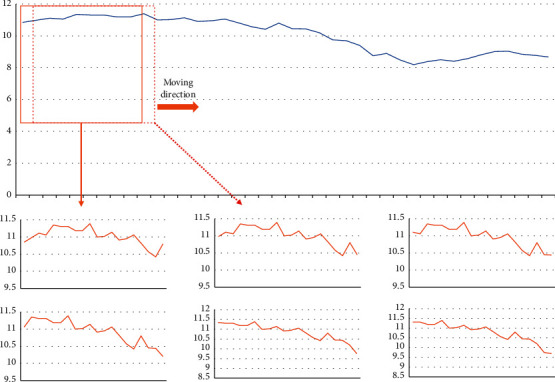
Moving window method for stock data processing.

The moving window approach is the most often used time series analysis method. Suppose there is a time series: *x*={*x*_*i*_*|x*_*i*_ ∈ *R*, *i*=1,2,…, *L*}, and the last *m* values in the series should be predicted by the first *n* data in the series. The first *n* data predictions are used as a moving window to map the value of the future time. Specifically, suppose we use the first 20 data to predict the 21st data for the first time, then, next, we add the 21st data from the previous prediction to the moving window, and delete the first data in the moving window. As a result, there are 20 new pieces of data in the window to anticipate the next data, and so on. To utilize the moving window approach, remove and add continually. When the window size is 20 [19–21], Figure 1 depicts a schematic representation of processing portion of stock data using the moving window approach.

Furthermore, data normalization is particularly crucial in data processing and subsequent studies, because different stock data is used as input for various perspectives. Take closing price and transaction volume as an example. When these two types of data are input as feature values, the value range is quite different. It is necessary to avoid excessively large transaction volume from having too much influence on the forecast result, so the data needs to be normalized. Therefore, data normalization can control the range of the data within a reasonable range [22].

### 2.2. RBF NN Model

**Figure 2:**
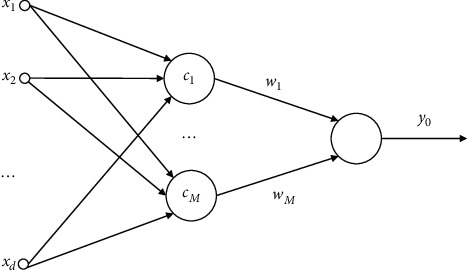
Classical RBF NN model structure.

Figure 2 depicts the standard RBF NN model. The RBF NN is a single-hidden-layer forward network. The input layer is the first one. The concealed layer is the second layer. The number of nodes in the hidden layer is determined by the problem's requirements. The radial basis function (RBF), also known as the transformation function, of the buried layer neurons is a nonnegative linear function that is radially symmetric and attenuated to the center point. The low-dimensional pattern input data is translated into the high-dimensional space, and the input vector is changed into the high-dimensional space, so that the linearly inseparable problem in the low-dimensional space becomes linearly separable in the high-dimensional space. The third layer is the output layer, which reacts to input patterns [23].

In the RBF NN model, the *f* : *R*^*d*^⟶*R*^1^ is the nonlinear mapping, and it can be expressed mathematically as

(1) $$\begin{array}{c} y^{0}=f\left(x\right)\\ =\sum_{i=1}^{M}\omega_{i}\Phi \left(\left\|x-c_{i}\right\|\right),\;i=1,\ldots ,M, \end{array}$$

where *x* ∈ *R*^*d*^ is the network's input vector, and *c*_*i*_ ∈ *R*^*d*^ is the center vector of the network's hidden layer nodes' RBF NN. The connection weight between the network's hidden layer and the output node is *ω*_*j*_. The norm is represented by ·, and here is the Euclidean norm. The RBF NN Φ(*·*) completes the nonlinear transformation of *R*^*d*^⟶*R*^1^ and has the following form:

(2) $$\begin{array}{c} \Phi \left(x-c_{i}\right)=\exp\left(-\frac{x-c_{i}^{2}}{\delta_{i}}\right), \end{array}$$

where *δ*_*i*_ is the width value.

Three parameters are required in the RBF NN model presented above. The hidden layer center node *c*_*i*_=[*c*_*i*_^1^,…,*c*_*i*_^*k*^,…,*c*_*i*_^*d*^]^*T*^, the hidden layer width value *δ*_*i*_, the hidden layer connection weight, and the output layer *ω*_*i*_.

In the RBF NN model described above, three parameters are needed; namely, the hidden layer center node *c*_*i*_=[*c*_*i*_^1^,…,*c*_*i*_^*k*^,…,*c*_*i*_^*d*^]^*T*^, the width value of the hidden layer *δ*_*i*_, and the connection weight of the hidden layer and the output layer *ω*_*i*_.

### 2.3. RBF NN with Linear Model

The central node parameter *c*_*i*_=[*c*_*i*_^1^,…,*c*_*i*_^*k*^,…,*c*_*i*_^*d*^]^*T*^ is estimated using the clustering approach, and the RBF NN's hidden layer width parameter is *δ*_*i*_. The fuzzy C-means (FCM) clustering algorithm is used in this work [24], and *c*_*i*_=[*c*_*i*_^1^,…,*c*_*i*_^*k*^,…,*c*_*i*_^*d*^]^*T*^ and *δ*_*i*_ can be estimated by the following formula:

(3) $$\begin{array}{c} c_{ik}=\frac{\sum_{j=1}^{N}u_{ji}x_{jk}}{\sum_{j=1}^{N}u_{ji}}, \end{array}$$

(4) $$\begin{array}{c} \delta_{i}^{k}=h\frac{\sum_{j=1}^{N}u_{ji}{\left\|x-c_{i}\right\|}^{2}}{\sum_{j=1}^{N}u_{ji}}, \end{array}$$

where *u*_*ji*_ represents the fuzzy membership of the sample *x*_*j*_=[*x*_*j*1_,…,*x*_*jd*_]^*T*^ calculated by the FCM clustering method to the  *i*-th category [25–27], and the parameter *h* is an adjustable scaling parameter.

If the hidden layer parameters of RBF NN are computed using the aforementioned clustering approach, then there are [8]

(5) $$\begin{array}{c} {\tilde{x}}^{i}=\Phi \left(x-c_{i}\right),\;i=1,\ldots ,M, \end{array}$$

(6) $$\begin{array}{c} x_{g}={\left[{\tilde{x}}^{1},{\tilde{x}}^{2},\ldots ,{\tilde{x}}^{M}\right]}^{T}, \end{array}$$

(7) $$\begin{array}{c} P_{g}={\left[\omega_{1},\ldots ,\omega_{M}\right]}^{T}. \end{array}$$

The radial basis network's mapping function, presented in formula (1), may be written as

(8) $$\begin{array}{c} y^{0}=p_{g}^{T}x_{g}. \end{array}$$

When the hidden layer nodes of the radial basis network are computed using the clustering approach, the network's final output may be described as the output of a linear model using formula (8). In this way, the parameter learning process of the network is transformed into a parameter learning problem of a linear model. Based on this linear model, the *ε*-insensitive loss function is introduced to construct a multiview learning item, so that the radial basis network can meet the needs of multiview learning.

### 2.4. RBF NN Using *ε*-Insensitive Loss Function

The RBF NN's objective function, based on the *ε*-insensitive loss function, is defined as

(9) $$\begin{array}{c} \left.\begin{array}{cc} \underset{P_{g}}{\min} & L=\sum_{i=1}^{N}\left|y_{i}^{0}-y_{i}\right|=\sum_{i=1}^{N}{\left|P_{g}^{T}x_{gi}-y_{i}\right|}_{\varepsilon }\\ \text{s.t. } & \left\{\begin{array}{c} y_{i}-P_{g}^{T}x_{gi}<\varepsilon +\xi_{i}^{+}\\ P_{g}^{T}x_{gi}-y_{i}<\varepsilon +\xi_{i}^{-} \end{array},\forall i\right.. \end{array}\right\}. \end{array}$$

By introducing slack variables *ξ*_*i*_^+^ and *ξ*_*i*_^−^, using the theory and method of quadratic programming, formula (9) may be rewritten as a standard quadratic programming problem, with the following particular form:

(10) $$\begin{array}{c} \left.\begin{array}{cc} \underset{P_{g},\xi^{+},\xi^{-},\varepsilon }{\text{min}} & L\left(P_{g},\xi^{+},\xi^{-},\varepsilon \right)=\frac{1}{\tau }\cdot \frac{1}{N}\overset{N}{\underset{i=1}{\sum }}\left({\left(\xi_{i}^{+}\right)}^{2}+{\left(\xi_{i}^{-}\right)}^{2}\right)+\frac{1}{2}\left(P_{g}^{T}P_{g}\right)+\frac{2}{\tau }\cdot \varepsilon \\ \text{s.t.} & \left\{\begin{array}{c} y_{i}-P_{g}^{T}x_{gi}<\varepsilon +\xi_{i}^{+}\\ P_{g}^{T}x_{gi}-y_{i}<\varepsilon +\xi_{i}^{-} \end{array},\forall i\right.. \end{array}\right\}. \end{array}$$

Formula (10) introduces the Lagrange multiplier term, transforming formula (9) into the quadratic programming problem of formula (11),

(11) $$\begin{array}{c} \left.\begin{array}{cc} \underset{\tilde{\alpha }}{\text{arg max}} & -{\tilde{\alpha }}^{T}\tilde{H}\tilde{\alpha }+{\tilde{\alpha }}^{T}\beta \\ \text{s.t.} & {\tilde{\alpha }}^{T}=1,\;\alpha_{i}\geq 0,\;\forall i, \end{array}\right\}, \end{array}$$

where $\tilde{H}={\left[{\tilde{h}}_{ij}\right]}_{2N\times 2N}$, $\;{\tilde{h}}_{ij}=z_{i}^{T}z_{i}+\left(\left(N\tau \right)/2\right)\delta_{ij}$, and $\delta_{ij}=\left\{\begin{array}{cc} 1 & i=j\\ 0 & i\neq j \end{array}\right.$.

The optimal solution of the parameters (*λ*^+^)^*∗*^ and(*λ*^−^)^*∗*^ can be achieved by solving formula (10). To obtain the best solution of the generalization term *P*_*g*_ of the RBF NN based on thee *ε*-insensitive loss function, use formulas (12a) and (12d).

(12a) $$\begin{array}{c} P_{g}=\frac{2}{\tau }\sum_{i=1}^{N}\left(\lambda_{i}^{+}-\lambda_{i}^{-}\right)x_{gi}, \end{array}$$

(12b) $$\begin{array}{c} \xi_{i}^{+}=N\lambda_{i}^{+}, \end{array}$$

(12c) $$\begin{array}{c} \xi_{i}^{-}=N\lambda_{i}^{-}, \end{array}$$

(12d) $$\begin{array}{c} \varepsilon =\sum_{i=1}^{N}\left(\lambda_{i}^{+}-\lambda_{i}^{-}\right)x_{gi}-\frac{N}{2}\left({\left(\lambda_{i}^{+}\right)}^{2}+{\left(\lambda_{i}^{-}\right)}^{2}\right)-\frac{1}{\tau }\sum_{i=1}^{N}\sum_{j=1}^{N}\left(\lambda_{i}^{+}-\lambda_{i}^{-}\right)\left(\lambda_{j}^{+}-\lambda_{j}^{-}\right)\cdot x_{gi}^{T}x_{gj}. \end{array}$$

The resultant parameters of the RBF NN based on the *ε*-insensitive loss function can be generated using the antecedent parameters, such as the hidden layer center node and width parameters, calculated using formulas (3) and (4), in combination with the subsequent parameters calculated using formulas (12a) and (12d).

## 3. Multiview RBF NN Model Framework

A single-view data collection is typically used to build traditional RBF neural networks. For example, previous closing prices are utilized as training data to anticipate the rise and fall of the next trading day when predicting stock price trends. However, while each trading day generates many stock attribute data, only the closing price is utilized as training data, and the impact of other attribute data on prediction outcomes cannot be accounted for in the prediction model. When the traditional RBF network is dealing with a data set composed of multiple data (multiview), a more feasible strategy is to use samples from different views to construct a corresponding network model. This strategy provides a feasible solution for applications in multiview scenarios, but the correlation between the views cannot be reflected in this strategy. This will also lead to uneven generalization capabilities of prediction models obtained from different views.

**Figure 3:**
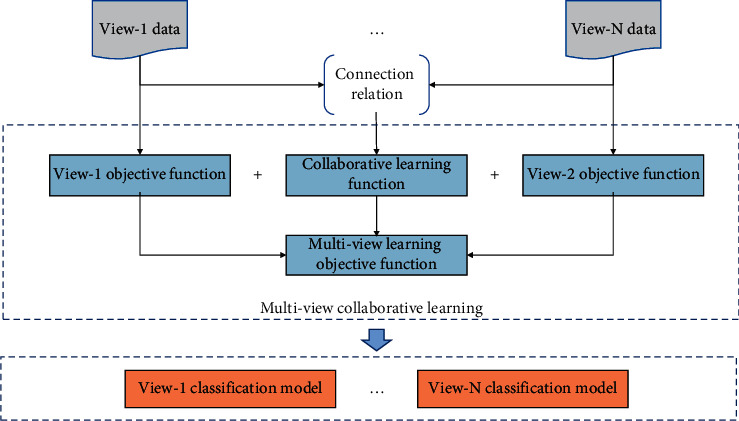
Multiview RBF classification model learning framework.

This study proposes a modeling notion as illustrated in Figure 3 to express the correlation between data from distinct viewpoints in the model's prediction process, so that the new model may learn from numerous views. In this modeling strategy, data from multiple views are learned collaboratively, instead of training in isolation of sample data from each view. Such a strategy can improve the generalization ability of the new model for data from various views and improve the applicability of the model.

**Figure 4:**
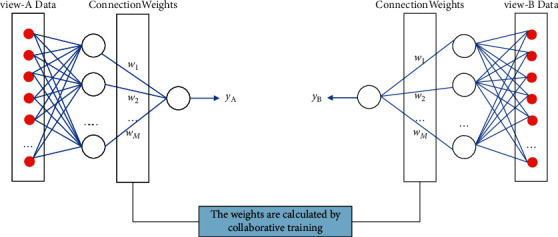
Data Processing in the model.

Figure 4 depicts the overall data input method into the multiview RBF model. The data from views A and B are entered into the RBF model corresponding to view A and view B, respectively, after preprocessing. The connection weight between the hidden layer and the output layer is defined by (*P*_*g*,*A*_)^*∗*^ and (*P*_*g*,*B*_)^*∗*^ calculated by the following training process in the RBF model's three-layer structure.

### 3.1. The Construction of Multiview Collaborative Learning Objective Function

The RBF NN is one of the most successful modeling approaches currently available. The least square error criteria are used to implement the standard RBF neural network. This sort of training strategy is best for small sample data sets or noisy data sets, as it is easy to overfit, reducing the RBF neural network's generalization capabilities. In order to address this issue, this work develops a new objective function by including the *ε*-insensitive loss function and structural risk term and then converts the suggested new objective function's solution into a standard quadratic programming problem. This successfully overcomes the overfitting problem as well as noise sensitivity flaws, while also demonstrating strong resilience.

In this section, dual views are taken as the specific research scene. Based on the traditional RBF classification model, the construction of the objective function of the multiview RBF classification model is discussed. To make full use of the independence of samples between different views and the correlation between data from different views while building a multiview RBF classification model, this study provides the following objective function formula with multiview learning capabilities.

(13) $$\begin{array}{c} \left.\begin{array}{cc} \underset{P_{g},\xi^{+},\xi^{-},\varepsilon }{\text{Min}} & l_{A}\left(P_{g,A},\xi_{A}^{+},\xi_{A}^{-},\varepsilon_{A}\right)+l_{B}\left(P_{g,B},\xi_{B}^{+},\xi_{B}^{-},\varepsilon_{B}\right)+l_{S}\left(\eta^{+},\eta^{-},\varepsilon_{S}\right)\\ \text{s.t.} & \left\{\begin{array}{c} y_{i,A}-P_{g,A}^{T}x_{gi,A}<\varepsilon_{A}+\xi_{i,A}^{+}\\ P_{g,A}^{T}x_{gi,A}-y_{i,A}<\varepsilon_{A}+\xi_{i,A}^{-}\\ y_{i,B}-P_{g,B}^{T}x_{gi,B}<\varepsilon_{B}+\xi_{i,B}^{+}\\ P_{g,B}^{T}x_{gi,B}-y_{i,B}<\varepsilon_{B}+\xi_{i,B}^{+}\\ P_{g,B}^{T}x_{gi,B}-P_{g,A}^{T}x_{gi,A}<\varepsilon_{S}+\eta_{i}^{+}\\ P_{g,A}^{T}x_{gi,A}-P_{g,B}^{T}x_{gi,B}<\varepsilon_{S}+\eta_{i}^{-} \end{array}\right.,\forall i, \end{array}\right\}. \end{array}$$

where

(13a) $$\begin{array}{c} l_{A}\left(P_{g,A},\xi_{A}^{+},\xi_{A}^{-},\varepsilon_{A}\right)=\frac{1}{N\tau_{A}}\sum_{i=1}^{N}\left[{\left(\xi_{i,A}^{+}\right)}^{2}+{\left(\xi_{i,A}^{-}\right)}^{2}\right]+\frac{1}{2}\left(P_{g,A}^{T}P_{g,A}\right)+\frac{2}{\tau_{A}}\cdot \varepsilon_{A}, \end{array}$$

(13b) $$\begin{array}{c} l_{B}\left(P_{g,B},\xi_{B}^{+},\xi_{B}^{-},\varepsilon_{B}\right)=\frac{1}{N\tau_{B}}\sum_{i=1}^{N}\left[{\left(\xi_{i,B}^{+}\right)}^{2}+{\left(\xi_{i,B}^{-}\right)}^{2}\right]+\frac{1}{2}\left(P_{g,B}^{T}P_{g,B}\right)+\frac{2}{\tau_{B}}\cdot \varepsilon_{B}, \end{array}$$

(13c) $$\begin{array}{c} l_{S}\left(\eta^{+},\eta^{-},\varepsilon_{S}\right)=\frac{1}{N\tau_{S}}\sum_{i=1}^{N}\left[{\left(\eta_{i}^{+}\right)}^{2}+{\left(\eta_{i}^{-}\right)}^{2}\right]+\frac{2}{\tau_{S}}\cdot \varepsilon_{S}. \end{array}$$

In formula (13), *l*_*A*_(*P*_*g*,*A*_, *ξ*_*A*_^+^, *ξ*_*A*_^−^, *ε*_*A*_) and *l*_*B*_(*P*_*g*,*B*_, *ξ*_*B*_^+^, *ξ*_*B*_^−^, *ε*_*B*_) are the objective function terms of the RBF classification model established based on the data samples of two independent views A and B. These two ensure that new approaches and strategies may take full use of variances in the characteristics of data samples from different perspectives, resulting in a classification model that is specific to the view's features.

The new modeling technique takes into account not only the changes in sample features between views (independent information), but also the capacity of multiview learning to discover the link between views, i.e., the consistency of each view's decision output. This study creates formula (13c) with collaborative learning capacity in response to such demands. Formula (13c) can make view A and view B undertake collaborative learning. Finally, the output of the classification model corresponding to each view tends to be consistent, and the difference in the generalization performance of the classification model of each view caused by the change of perspective feature is avoided.

It should be noted that the regularization parameter *τ*_*A*_ > 0,  *τ*_*B*_ > 0,  *τ*_*S*_ > 0 controls the complexity and degree of error of the classification model, and its value is manually set to select an appropriate value [28–30].

### 3.2. MV-RBF Parameter Learning Rules

Continuing to derive the objective function formula, the Lagrange equation is as follows:

(14) $$\begin{array}{c} J=\frac{1}{N\tau_{A}}\sum_{i=1}^{N}\left[{\left(\xi_{i,A}^{+}\right)}^{2}+{\left(\xi_{i,A}^{-}\right)}^{2}\right]+\frac{1}{2}\left(P_{g,A}^{T}P_{g,A}\right)+\frac{2}{\tau_{A}}\cdot \varepsilon_{A}+\\ \frac{1}{N\tau_{B}}\sum_{i=1}^{N}\left[{\left(\xi_{i,B}^{+}\right)}^{2}+{\left(\xi_{i,B}^{-}\right)}^{2}\right]+\frac{1}{2}\left(P_{g,B}^{T}P_{g,B}\right)+\frac{2}{\tau_{B}}\cdot \varepsilon_{B}+\frac{1}{N\tau_{S}}\sum_{i=1}^{N}\left[{\left(\eta_{i}^{+}\right)}^{2}+{\left(\eta_{i}^{-}\right)}^{2}\right]\\ +\frac{2}{\tau_{S}}\cdot \varepsilon_{S}+\sum_{i=1}^{N}\lambda_{i,A}^{+}\left(y_{i,A}-P_{g,A}^{T}x_{gi,A}-\varepsilon_{A}-\xi_{i,A}^{+}\right)+\sum_{i=1}^{N}\lambda_{i,A}^{-}\left(P_{g,A}^{T}x_{gi,A}-y_{i,A}-\varepsilon_{A}-\xi_{i,A}^{+}\right)\\ +\sum_{i=1}^{N}\lambda_{i,B}^{N}\left(y_{i,B}-P_{g,B}^{T}x_{gi,B}-\varepsilon_{B}-\xi_{i,B}^{+}\right)+\sum_{i=1}^{N}\lambda_{i,A}^{-}\left(P_{g,B}^{T}x_{gi,B}-y_{i,B}-\varepsilon_{B}-\xi_{i,B}^{+}\right)+\\ \sum_{i=1}^{N}\beta_{i}^{+}\left(P_{g,B}^{T}x_{gi,B}-P_{g,A}^{T}x_{gi,A}-\varepsilon_{S}-\eta_{i}^{+}\right)+\sum_{i=1}^{N}\beta_{i}^{-}\left(P_{g,A}^{T}x_{gi,A}-P_{g,B}^{T}x_{gi,B}-\varepsilon_{S}-\eta_{i}^{-}\right). \end{array}$$

According to the partial derivative of the variable in equation (14) being 0, the following relationship can be derived:

(15) $$\begin{array}{c} P_{g,A}=\sum_{i=1}^{N}\left(\lambda_{i,A}^{+}-\lambda_{i,A}^{-}\right)x_{gi,A}+\sum_{i=1}^{N}\left(\beta_{i,S}^{+}-\beta_{i,S}^{-}\right)x_{gi,A}, \end{array}$$

(16) $$\begin{array}{c} P_{g,B}=\sum_{i=1}^{N}\left(\lambda_{i,B}^{+}-\lambda_{i,B}^{-}\right)x_{gi,B}+\sum_{i=1}^{N}\left(\beta_{i,S}^{+}-\beta_{i,S}^{-}\right)x_{gi,B}, \end{array}$$

(17) $$\begin{array}{c} \xi_{i,A}^{+}=\frac{N\tau_{A}}{2}\lambda_{i,A}^{+}, \end{array}$$

(18) $$\begin{array}{c} \xi_{i,A}^{-}=\frac{N\tau_{A}}{2}\lambda_{i,A}^{-}, \end{array}$$

(19) $$\begin{array}{c} \xi_{i,B}^{+}=\frac{N\tau_{B}}{2}\lambda_{i,B}^{+}, \end{array}$$

(20) $$\begin{array}{c} \xi_{i,B}^{-}=\frac{N\tau_{B}}{2}\lambda_{i,B}^{-}, \end{array}$$

(21) $$\begin{array}{c} \eta_{i}^{+}=\frac{N\tau_{S}}{2}\beta_{i}^{+}, \end{array}$$

(22) $$\begin{array}{c} \eta_{i}^{-}=\frac{N\tau_{S}}{2}\beta_{i}^{-}, \end{array}$$

(23) $$\begin{array}{c} \frac{2}{\tau_{A}}=\sum_{i=1}^{N}\left(\lambda_{i,A}^{+}-\lambda_{i,A}^{-}\right), \end{array}$$

(24) $$\begin{array}{c} \frac{2}{\tau_{B}}=\sum_{i=1}^{N}\left(\lambda_{i,B}^{+}-\lambda_{i,B}^{-}\right), \end{array}$$

(25) $$\begin{array}{c} \frac{2}{\tau_{S}}=\sum_{i=1}^{N}\left(\beta_{i}^{+}-\beta_{i}^{-}\right). \end{array}$$

Substituting formula (15) to (25) into (14), and deriving formula (26) after simplification,

(26) $$\begin{array}{c} \left.\begin{array}{cc} \underset{\alpha }{\text{arg max}} & -\frac{1}{2}\alpha^{T}H\alpha +\alpha^{T}\beta \\ \text{s.t. } & \left\{\begin{array}{c} \alpha^{T}L_{A}=\frac{2}{\tau_{A}},\\ \;L_{A}=\left[\underset{2N}{1,\ldots ,1},\underset{2N}{0,\ldots ,0},\underset{2N}{0,\ldots ,0}\right]\\ \alpha^{T}L_{B}=\frac{2}{\tau_{B}},\\ \;L_{B}=\left[\underset{2N}{0,\ldots ,0},\underset{2N}{1,\ldots ,1},\underset{2N}{0,\ldots ,0}\right],\;\alpha_{i}\geq 0,\forall_{i}\\ \alpha^{T}L_{S}=\frac{2}{\tau_{B}},\\ \;L_{B}=\left[\underset{2N}{0,\ldots ,0},\underset{2N}{0,\ldots ,0},\underset{2N}{1,\ldots ,1}\right], \end{array}\right. \end{array}\right\}. \end{array}$$

where

(26a) $$\begin{array}{c} {\tilde{H}}_{A}={\left[{\tilde{h}}_{ij,A}\right]}_{2N\times 2N},\\ \;{\tilde{h}}_{ij,A}=z_{i,A}^{T}z_{i,A}+\left(\frac{\left(N\tau_{A}\right)}{2}\right)\delta_{ij,A},\;\\ \delta_{ij,A}=\left\{\begin{array}{cc} 1, & \;i=j,\\ 0, & i\neq j, \end{array}\right. \end{array}$$

(26b) $$\begin{array}{c} {\tilde{H}}_{B}={\left[{\tilde{h}}_{ij,B}\right]}_{2N\times 2N},\\ \;{\tilde{h}}_{ij,B}=z_{i,B}^{T}z_{i,B}+\left(\frac{\left(N\tau_{B}\right)}{2}\right)\delta_{ij,B},\;\\ \delta_{ij,B}=\left\{\begin{array}{cc} 1, & \;i=j,\\ 0, & i\neq j, \end{array}\right.\\ {\tilde{h}}_{ij,S}=-3z_{i,B}^{T}z_{i,B}+z_{i,A}^{T}z_{i,A}+\left(\frac{\left(N\tau_{S}\right)}{2}\right)\delta_{ij,S},\;\\ \delta_{ij,S}=\left\{\begin{array}{cc} 1, & \;i=j,\\ 0, & i\neq j, \end{array}\right.\\ {\tilde{h}}_{ij,AS}=z_{i,A}^{T}z_{i,A},\\ {\tilde{h}}_{ij,BS}=z_{i,B}^{T}z_{i,B}, \end{array}$$

(26c) $$\begin{array}{c} {\tilde{H}}_{S}={\left[{\tilde{h}}_{ij,S}\right]}_{2N\times 2N},\;\\ {\tilde{h}}_{ij,S}=-3z_{i,B}^{T}z_{i,B}+z_{i,A}^{T}z_{i,A}+\left(\frac{\left(N\tau_{S}\right)}{2}\right)\delta_{ij,S},\;\\ \delta_{ij,S}=\left\{\begin{array}{cc} 1, & \;i=j,\\ 0, & i\neq j, \end{array}\right.\\ {\tilde{h}}_{ij,AS}=z_{i,A}^{T}z_{i,A},\\ {\tilde{h}}_{ij,BS}=z_{i,B}^{T}z_{i,B}, \end{array}$$

(26d) $$\begin{array}{c} {\tilde{H}}_{AS}={\left[{\tilde{h}}_{ij,AS}\right]}_{2N\times 2N},\\ {\tilde{h}}_{ij,AS}=z_{i,A}^{T}z_{i,A},\\ {\tilde{h}}_{ij,BS}=z_{i,B}^{T}z_{i,B}, \end{array}$$

(26e) $$\begin{array}{c} {\tilde{H}}_{BS}={\left[{\tilde{h}}_{ij,BS}\right]}_{2N\times 2N},\\ {\tilde{h}}_{ij,BS}=z_{i,B}^{T}z_{i,B}, \end{array}$$

(26f) $$\begin{array}{c} H=\left(\begin{array}{ccc} {\tilde{H}}_{A} & 0 & {\tilde{H}}_{AS}\\ 0 & {\tilde{H}}_{B} & -{\tilde{H}}_{BS}\\ {\tilde{H}}_{AS} & -{\tilde{H}}_{BS} & {\tilde{H}}_{S} \end{array}\right). \end{array}$$

At this point, formula (14) has been transformed into a classic quadratic programming problem as shown in formulas (26), and (26) is solved using the quadratic programming solution method in literature [8]. After completing the quadratic programming calculation, the optimized parameters (*λ*_*A*_^+^)^*∗*^， (*λ*_*A*_^−^)^*∗*^， (*λ*_*B*_^+^)^*∗*^， (*λ*_*B*_^−^)^*∗*^， (*β*_*S*_^+^)^*∗*^， (*β*_*S*_^−^)^*∗*^ corresponding to formula (26) are obtained. Using these optimized parameters, we can derive the subsequent parameters of the optimized RBF classification model under each view as follows:

(27a) $$\begin{array}{c} {\left(P_{g,A}\right)}^{*}=\sum_{i=1}^{N}\left({\left(\lambda_{i,A}^{+}\right)}^{*}-{\left(\lambda_{i,A}^{-}\right)}^{*}\right)x_{gi,A}+\sum_{i=1}^{N}\left({\left(\beta_{i,S}^{+}\right)}^{*}-{\left(\beta_{i,S}^{-}\right)}^{*}\right)x_{gi,A}, \end{array}$$

(27b) $$\begin{array}{c} {\left(P_{g,B}\right)}^{*}=\sum_{i=1}^{N}\left({\left(\lambda_{i,B}^{+}\right)}^{*}-{\left(\lambda_{i,B}^{-}\right)}^{*}\right)x_{gi,B}+\sum_{i=1}^{N}\left({\left(\beta_{i,S}^{+}\right)}^{*}-{\left(\beta_{i,S}^{-}\right)}^{*}\right)x_{gi,B}. \end{array}$$

Formula (26a) is the subsequent parameter of the RBF classification model corresponding to view A, and formula (26b) is the subsequent parameter of the RBF classification model corresponding to view B. As mentioned above, the cluster center and width parameters calculated by formulas (3) and (4) are used as the antecedent parameters of the multiview RBF classification model. This gives us access to all of the parameters that make up the multiview RBF classification model.  Algorithm 1 depicts the multiview RBF classification model (MV-RBF) learning algorithm; please see the detailed Algorithm 1 as follows:

## 4. Experiment

In this part, we create two sets of experiments to test the performance of the multiview RBF classification model in stock trend prediction and compare and contrast the multiview RBF classification model to other algorithm models. To prove MV practicality, RBF's first set of trials compares it to the classic RBF classification model. The second set of trials compares MV-RBF against a well-used classification technique.

### 4.1. Setup

To verify the feasibility of the multiview RBF classification model (MV-RBF) proposed in this paper, this section selects Lvjing real estate (ID 000502) from January 2, 2018, to December 31, 2019, for a total of 473 trading days. The various indicators constitute the original data set. We picked the two characteristics of the closing price and volume in the original data set as the two perspectives of the experiment and processed them into two data sets *D*_1_ and *D*_2_ using the moving window approach and data normalization described in the previous study. The data sets *D*_1_ and *D*_2_ have a window size of 20, which means that the input data has a dimension of 20. Part of the data in the data set for data specification is shown in Table 1.

Table 1 shows part of the data in a single-view data set. Each row is used as an input, and the last column of each row represents the fluctuation of the predicted trading day and also serves as the two categories of the model classification. In addition, we changed the size of the moving window, that is, the dimension of the input data, to find a suitable window size to improve the accuracy of classification. We also chose two other features from the original data set as two perspectives, such as opening and closing prices, opening and highest prices, to see how the choice of view affected the categorization findings.

Some symbolic representations involved in the experiment are defined as follows: *D*_1_ and *D*_2_ represent the training data set of view 1 and view 2 in the multiview scene, respectively, and *D*_1__test, *D*_2__test are the test data sets. [*D*_1_,  *D*_2_] represents the single-view training data set obtained by combining the training data sets of view 1 and view 2 by the feature expansion method, and [*D*_1_,  *D*_2_]_test is the test data sets. RBF (View-*D*_1_), RBF (View-*D*_2_) and RBF (View-[*D*_1_,  *D*_2_]) are classification models constructed by traditional RBF neural networks based on the *D*_1_, *D*_2_ and [*D*_1_,  *D*_2_] data sets. The multiview RBF neural network modeling techniques MV-RBF (View-*D*_1_) and MV-RBF (View-*D*_2_) suggested in this study are based on the classification model generated by *D*_1_ and *D*_2_.

Since this paper is to predict the rise and fall of stock prices, we choose the accuracy that is often used in classification tasks. The accuracy rate can reflect the proportion of the correct prediction in the test sample.

The manual setting parameters involved in the experiment process include regularization parameters *τ*_*A*_,  *τ*_*B*_,  *τ*_*S*_ and the number of hidden layer nodes of the RBF neural network *M*, all of which use the parameter ranges given by cross-validation in Table 2.

B. Comparison of MV-RBF and traditional RBF classification model.

We used the moving window method and data normalization to standardize the form of the data set in this section of the experiment. We selected the closing price and volume data as the experimental data sets *D*_1_ and *D*_2_ and used the moving window method and data normalization to standardize the form of the data set. We used a basic cross-validation procedure, scrambled the data sets *D*_1_ and *D*_2_, and ran many trials to further demonstrate the practicality of MV-RBF.  Table 3 shows the outcomes of the experiment.

Six sets of experimental data are shown in Table 3. The classification impact of the multiview RBF model is higher than that of the classic RBF model, according to the experimental data. This demonstrates that when compared to the traditional single-view method, the method of constructing multiview in this paper has the ability of multiview learning, as it can more effectively utilize the independent feature space information in the data set of each view and the correlation between views. The effectiveness of the classification approach used in this study may be validated.

The 6 groups of experimental results of the traditional RBF classification model on 3 data sets in Table 3 can show that the single-view sample [*D*_1_,  *D*_2_] artificially constructed by the feature expansion method can balance the difference between views to a certain extent. This shows that although there is a certain correlation between the various views, the differences between the views are also obvious. In most circumstances, the classification effect of the single-view sample [*D*_1_,  *D*_2_] based on the standard RBF model is lower than the classification effect of the classification model based on the technique of this study in any view due to this disparity.

During the experiment, we also encountered some other situations. In the fourth set of experiments in Table 3, the classification accuracy of the view *D*_1_ dataset under the two classification models is the same. No matter how the parameters of MV-RBF are adjusted, the accuracy will not be significantly improved. In addition, in the experimental group not shown in Table 3, there are similar situations where the classification effects of the two models for the same data set are similar. With such a result, we infer that the two data sets failed to provide useful spatial feature information for each other in the process of collaborative learning, or the data sets of the two views selected in the experiment are data of two similar attributes of stocks. Even if the collaborative training process is carried out, due to the similar attributes, there is not much correlation between the two data sets, resulting in the fact that the model classification effect is not much improved.

### 4.2. Comparison of Multiview RBF Classification Model with Other Commonly Used Classification Models

In the preceding part, we compared the multiview method's classification impact to that of the classic RBF classification model, demonstrating the multiview RBF classification model's efficacy. In this part, we compare the multiview approach presented in this research against other regularly used categorization methods in order to confirm its viability. For comparison, we used three frequently used classification methods: decision tree, support vector machine (SVM), and closest neighbor classifier (KNN), and the experimental results are displayed in Table 4.

The experimental findings in Table 4 show that the multiview RBF classification model presented in this research can be used for stock prediction analysis in the vast majority of situations. Except for individual studies, the MV-RBF model has a somewhat greater classification impact than other regularly used classification models. This also demonstrates that, during model training, the RBF classification approach with multiview learning capability can generate the classification model for each view by fully using the independent component and relevance component of the multiview data set. Whether we look at these trained models from an independent single point of view or the correlation between several points of view, the results suggest that the strategy in this research is feasible and successful. As a result, the classification model created using the approach described in this study may forecast stock price trends and can improve the classification impact of the single-view classification model.

### 4.3. Experimental Result of MV-RBF Classification Model on Other Data Sets

To further validate the efficiency of the strategy in this research, we picked stock data from different industries and sectors and processed them into acceptable data sets for experimentation.  Table 5 shows the experimental findings of the SAIC Motor stock data set with the stock code 600104, while Table 5 shows the experimental results of the GREE Electric stock data set with the stock code ID 000651.

The aforementioned experimental findings on various data sets demonstrate that the MV-RBF classification model performs better than the classic RBF classification model, demonstrating the efficacy of the strategy presented in this study.

### 4.4. Parameter Sensitivity Analysis and Cross-Validation to Determine the Approximate Range of Parameters

We employed the approach of controlled variables to examine the sensitivity of several parameters in the MV-RBF model in this section of the experiment. The number of nodes *M* in the RBF NN's hidden layer, and the regularization parameters *τ*_*A*_,  *τ*_*B*_,  *τ*_*S*_ in the multiview learning ability formula, are among these parameters. Compare the impact of adjusting these parameter values on the categorization model.  Table 6 illustrates the results of experiments with various numbers of hidden layer nodes.

**Figure 5:**
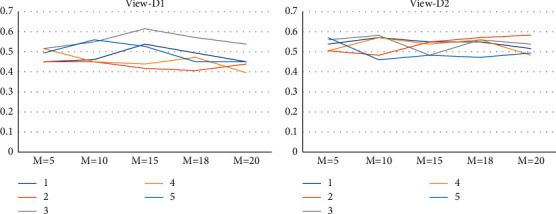
Parameter sensitivity line chart.

According to the experimental results in Table 6 and Figure 5, changing the number of hidden nodes in the RBF neural network had no significant effect on the experimental results, but it can be seen that the experimental data is roughly within a certain range after multiple sets of repeated experiments. This is because the RBF neural network's hidden layer employs the FCM clustering algorithm, which redivides the cluster centers with each trial; that is, it recalculates the relevant membership degree *u*_*ji*_ and the width parameter *h* (refer to Section 2). In addition, the regularization parameters *τ*_*A*_,  *τ*_*B*_,  *τ*_*S*_ in the multiperspective learning ability formula are also carried out in related experiments, and the experimental results are similar to Table 6. We find that the multiview RBF neural network classification model is less susceptible to parameters but has a specific active interval, thanks to the RBF neural network's hidden layer clustering mechanism.

There is currently no correct theoretical analysis or methodology for determining the ideal value of the insensitive parameter *ε*; however, this type of parameter is analogous to the insensitive parameter *ε* in the support vector regression (SVR) approach. The optimal value of this parameter and the variance of the noise in the data have an approximately proportional relationship, meaning that the greater the noise, the larger the value of this parameter usually required to obtain a good training effect, according to a theoretical study of this parameter in SVR.

The artificially set parameters *τ*_*A*_,  *τ*_*B*_,  *τ*_*S*_ involved in the experiment process control the complexity and degree of error of the classification model. The experimental results of finding the approximate range of the regularization parameters *τ*_*A*_,  *τ*_*B*_,  *τ*_*S*_ are shown in Table 7.

### 4.5. Complexity Analysis

The temporal complexity of the MV-RBF classification method is mostly made up of two parts, as illustrated in Algorithm 1: the learning of the antecedent parameters (corresponding to Step 2) and the learning of the subsequent parameters (corresponding to Steps 3 and 4). In multiview learning, the antecedent parameters of the classification model corresponding to each view of the MV-RBF algorithm are obtained by the FCM clustering algorithm, and its time complexity is O (*MTN*), where *M* is the number of views, *T* is the number of iterations in the algorithm, and *N* is the number of samples. The difficulty of determining the extreme value of quadratic programming is the key to learning the subsequent parameters of each MV-RBF view. The time complexity is O (*N*^3^). By using decomposition methods such as SMO to deal with secondary planning problems, it is possible to lower the time complexity to O (*N*^2^). Therefore, in multiview learning, the time complexity of MV-RBF subsequent parameter learning is between O (*MN*^2^)∼O (*MN*^3^). The traditional RBF classification model used in this paper has a time complexity of only O (*TN*) + O (*N*^2^), so the multiview MV-RBF algorithm is inferior to the traditional single-view RBF classification model in terms of time performance. The main reason is that the MV-RBF modeling strategy introduces a multiview collaborative learning method, while the traditional single-view RBF classification model does not have multiview learning capabilities, so the MV-RBF classification model is more time-consuming. However, the generalization performance of MV-RBF has a certain improvement compared with the traditional classification model.

## 5. Conclusion

This study builds on the standard single-view RBF classification model by including the concept of multiview collaborative learning and establishing a new multiview RBF classification model based on the independence and relevance information between the views. This approach successfully uses the independence component in the multiview data set as well as the correlation component between the views to improve the classification model's accuracy under each view.

The efficiency and practicality of the strategy in this research are demonstrated by a simulated experiment on a stock data set. However, owing to the complexity of the multiview methodology, this paper's study only covers the particular modeling approach of MV-RBF from a dual-view perspective. In the future, we will focus on developing a more compact and appropriate multiview categorization model. Simultaneously, the use of other traditional classification techniques in multiview scenarios will be examined.

## Figures and Tables

**Algorithm 1:**
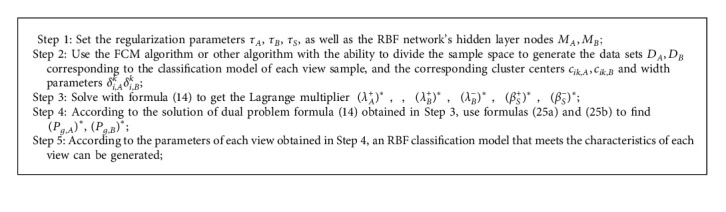
MV-RBF algorithm.

**Table 1:** A sample of the information in the data set.*D*_1_

	1	2	…	19	20	21
1	0.8841	0.9252	…	0.8766	0.8000	0
2	0.9252	0.9159	…	0.8000	0.7664	0
3	0.9159	0.9645	…	0.7664	0.7701	1
4	0.9645	0.9738	…	0.7701	0.6673	1
5	0.9738	0.9589	…	0.6673	0.6654	0

**Table 2:** Parameter settings of all classification models.

Models	Parameters	Search rangers
RBF	Number of hidden nodes M	{10, 11,…, 19,20}

MV-RBF	Regularization parameter *τ*_*A*_	{10^−3^, 10^−2^,…, 10^1^} or{2^0^, 2^1^,…, 2^4^}
	Regularization parameter *τ*_*B*_	{10^−3^, 10^−2^,…, 10^1^} or{2^0^, 2^1^,…, 2^4^}
	Regularization parameter *τ*_*S*_	{2^0^, 2^1^,…, 2^6^}
	Number of hidden nodes M	{10, 11,…, 19,20}

**Table 3:** A comparison of RBF and MV-RBF using accuracy index.

*D*_1_/*D*_2_	RBF		MV-RBF		
	*D*_1_	*D*_2_	[*D*_1_, *D*_2_]	*D*_1_	*D*_2_
1	0.5824	0.5495	0.5663	0.6154	0.5714
2	0.5055	0.5934	0.5862	0.5604	0.6044
3	0.5604	0.4615	0.5062	0.6044	0.5165
4	0.6923	0.4286	0.4941	0.6923	0.4945
5	0.5495	0.4615	0.5529	0.6484	0.5385
6	0.4954	0.4396	0.4494	0.5934	0.4725

**Table 4:** Comparison of accuracy between MV-RBF and other classification models.

	MV-RBF		Decision tree		Naive Bayes		SVM		KNN		Subspace KNN	
	The view of *D*_1_	The view of *D*_2_	The view of *D*_1_	The view of *D*_2_	The view of *D*_1_	The view of *D*_2_	The view of *D*_1_	The view of *D*_2_	The view of *D*_1_	The view of *D*_2_	The view of *D*_1_	The view of *D*_2_
1	0.6154	0.5714	0.6120	0.5240	0.5180	0.5460	0.5990	0.4890	0.4820	0.5040	0.4780	0.4890
2	0.5604	0.6044	0.5640	0.5480	0.5130	0.5310	0.6060	0.5200	0.5240	0.5110	0.4600	0.5130
3	0.6044	0.5165	0.5750	0.5510	0.5290	0.5480	0.5750	0.5590	0.5260	0.5150	0.4520	0.5180
4	0.6923	0.4945	0.6080	0.5550	0.4910	0.5310	0.5970	0.5330	0.5040	0.5180	0.4450	0.5240
5	0.6484	0.5385	0.5810	0.4760	0.4890	0.5480	0.5930	0.5200	0.4980	0.4980	0.4780	0.5020
6	0.5923	0.4725	0.5620	0.5260	0.5020	0.5350	0.6080	0.5510	0.4800	0.4850	0.4600	0.4850
7	0.5275	0.5385	0.6430	0.5240	0.5220	0.5310	0.5900	0.5460	0.4800	0.4820	0.4540	0.5240
8	0.6044	0.5055	0.5900	0.5200	0.5290	0.5510	0.5700	0.5460	0.4800	0.5020	0.4800	0.4910
9	0.6154	0.5165	0.6100	0.5400	0.5550	0.5400	0.6150	0.5150	0.5400	0.4890	0.4780	0.5000
10	0.5385	0.4835	0.5620	0.5370	0.5070	0.5570	0.5680	0.5680	0.5240	0.5290	0.4890	0.5370

**Table 5:** Experimental results on other data sets.

*D*_1_/*D*_2_	RBF		MV-RBF	
	The view of *D*_1_	The view of *D*_2_	The view of *D*_1_	The view of *D*_2_
1	0.5824	0.4176	0.6154	0.4835
2	0.5385	0.5385	0.5824	0.5495
3	0.6154	0.5165	0.6484	0.5495
4	0.4835	0.4725	0.5604	0.5495
5	0.4945	0.5934	0.5165	0.6154
6	0.5165	0.5604	0.5385	0.5714
7	0.4725	0.4615	0.5165	0.4395
8	0.4835	0.4286	0.5275	0.4615
9	0.5495	0.4945	0.5385	0.5604
10	0.4835	0.5495	0.5385	0.5714

**Table 6:** Parameter sensitivity analysis.

	*M*=5		*M*=10		*M*=15		*M*=18		*M*=20	
	*D*_1_	*D*_2_	*D*_1_	*D*_2_	*D*_1_	*D*_2_	*D*_1_	*D*_2_	*D*_1_	*D*_2_
1	0.4505	0.5385	0.4615	0.5714	0.5385	0.5495	0.4945	0.5495	0.4505	0.5165
2	0.4505	0.5055	0.4505	0.4835	0.4176	0.5495	0.4066	0.5714	0.4396	0.5835
3	0.5165	0.5604	0.5495	0.5824	0.6154	0.4835	0.5714	0.5604	0.5385	0.5385
4	0.5165	0.5055	0.4505	0.5714	0.4396	0.5385	0.4725	0.5604	0.3956	0.4835
5	0.4945	0.5714	0.5604	0.4604	0.5275	0.4835	0.4505	0.4725	0.4505	0.4945

**Table 7:** Cross-validation regularized parameter range.

*D*_1_/*D*_2_	*τ*_*A*_	*τ*_*B*_	*τ*_*S*_	*D*_1_	*D*_2_
1	10^0^	1	2^0^	0.5495	0.5055
	10^−1^	1	2^0^	0.5385	0.5275
	10^−2^	1	2^0^	0.5385	0.5495
	10^−3^	1	2^0^	0.5275	0.5385
	10^−4^	1	2^0^	0.5275	0.5385

2	1	10^0^	2^0^	0.5495	0.5165
	1	10^−1^	2^0^	0.5604	0.5165
	1	10^−2^	2^0^	0.5604	0.4945
	1	10^−3^	2^0^	0.5495	0.4835
	1	10^−4^	2^0^	0.5495	0.4835

3	1	1	2^0^	0.5495	0.5055
	1	1	2^1^	0.5275	0.5385
	1	1	2^2^	0.4945	0.5385
	1	1	2^3^	0.4945	0.5385
	1	1	2^4^	0.4945	0.5385

## Data Availability

The datasets used to support the findings of this study are available from the corresponding author upon request.

## References

[1] Murphy J. J. (1999). *Technical Analysis of the Financial Markets*.

[2] White H. Economic prediction using neural networks: the case of IBM daily stock returns.

[3] Zhang H. (2018). The forecasting model of stock price based on PCA and BP neural network. *Journal of Financial Risk Management*.

[4] Huang T. Y., Chen F. F. (2016). Application of kernel function of stock price forecasting based on SVM. *Journal of Chongqing University of Technology (Natural Science)*.

[5] Kim T., Kim H. Y. (2019). Forecasting stock prices with a feature fusion LSTM-CNN model using different representations of the same data. *PLoS One*.

[6] Moghar A., Hamiche M. (2020). Stock Market Prediction Using LSTM Recurrent Neural Network. *Procedia Computer Science*.

[7] Yizhang Jiang Y. Z., Fu-Lai Chung F. L., Shitong Wang S. T. (2015). Collaborative fuzzy clustering from multiple weighted views. *IEEE Transactions on Cybernetics*.

[8] Deng Z. H., Jiang Y. Z., Choi K. (2013). Knowledge-leverage-based TSK fuzzy system modeling. *IEEE Transactions on Neural Networks and Learning System*.

[9] Jiang Y. Z., Deng Z. H., Wang J. (2014). Collaborative partition multi-view fuzzy clustering algorithm using entropy weighting. *Journal of Software*.

[10] Zhang S. L., Yu X., Sui Y. (2015). Object tracking with multi-view support vector machines[J]. *IEEE Transactions on Multimedia*.

[11] Huang G. B., Zhu Q. Y., Siew C. K. (2006). Extreme learning machine: theory and applications. *Neurocomputing*.

[12] Lan Y., Soh Y. C., Huang G. B. (2010). Constructive hidden nodes selection of extreme learning machine for regression. *Neurocomputing*.

[13] Huang G. B., Ding X. J., Zhou H. M. (2011). Optimization method based extreme learning machine for classification. *Neurocomputing*.

[14] Zhang P., Zhou X., Pelliccione P. (2017). RBF-MLMR: a multi-label metamorphic relation prediction approach using RBF neural network. *IEEE Access*.

[15] Meng X., Rozycki P., Qiao J. F. (2018). Nonlinear system modeling using RBF networks for industrial application. *IEEE Transactions on Industrial Informatics*.

[16] Tzortzis G. F., Likas A. C. (2010). Multiple view clustering using a weighted combination of exemplar-based mixture models. *IEEE Transactions on Neural Networks*.

[17] Merugu S., Rosset S., Perlich C. A new multi-view regression approach with an application to customer wallet estimation.

[18] Li G. X., Chang K. Y., Hoi S. C. H. (2012). Multi-view semi-supervised learning with consensus. *IEEE Transactions on Knowledge and Data Engineering*.

[19] Zheng L., Hu W., Min Y. (2015). Raw wind data preprocessing: a data-mining approach. *IEEE Transactions on Sustainable Energy*.

[20] Zhang Z. B., Liu W. (2017). Analysis of data preprocessing technology in data mining. *Digital Technology and application*.

[21] Shi W., Zhu Y., Huang T., Sheng G., Lian Y., Wang G. (2017). An integrated data preprocessing framework based on Apache spark for fault diagnosis of power grid equipment. *Journal of Signal Processing System*.

[22] Zhan Z. Q., Zhao J. Y., Zhang Y., Gong J. T., Wang Q. Y. (2021). Grabbing the Long Tail: a data normalization method for diverse and information dialogue generation. *Neurocomputing*.

[23] Tao J. L., Yu Z., Zhang R. D., Gao F. R. (2021). RBF neural network modeling approach using PCA based LM-GA optimization for coke furnace system. *Applied Soft Computing*.

[24] Jiang Y. Z., Chung F. L., Wang S. T. (2014). Enhanced fuzzy partitions vs data randomness in FCM. *Journal of Intelligent and Fuzzy Systems*.

[25] Jang J. S. R., Sun C. T., Mizutani E. (1997). *Neuro-Fuzzy and Soft-Computing*.

[26] Vapnik V. (1998). *Statistical Learning Theory*.

[27] Wang S. T. (1998). *Neural Fuzzy System and its Application*.

[28] Ito K., Nakano R. Optimizing support vector regression hyperparameters based on cross-validation.

[29] Kwok J. T., Tsang I. W. (2003). Linear dependency between epsilon and the input noise in epsilon-support vector regression. *IEEE Transactions on Neural Networks*.

[30] Box G. E. P., Jenkins G. M. (1976). *Time Series Analysis, Forecasting and Control*.

